# Symptomatic therapy in ATTR amyloidosis: pain killers in TTR-FAP

**DOI:** 10.1186/1750-1172-10-S1-I1

**Published:** 2015-11-02

**Authors:** Nadine Attal

**Affiliations:** 1 INSERM U-987, Centre d'Evaluation et de Traitement de la Douleur, CHU Ambroise Parc APHP, F-92100 Boulogne-Billancourt, France and University Versailles Saint-Quentin, Versailles, F-78035, France

## 

Familial amyloidosis typically causes a nerve length-dependent small fiber polyneuropathy that starts in the feet with loss of temperature and pain sensations, associated with autonomic dysfunction, which can be extremely severe and life threatening. Neuropathic pain is commonly associated with amyloid neuropathy. There are no randomized controlled trials in peripheral neuropathies specifically related to familial amyloidosis. However meta-analyses have confirmed that the efficacy of drugs against neuropathic pain generally is not linked to the aetiology of pain and therefore the drugs found effective in other painful polyneuropathies may benefit to patients with amyloid neuropathies [1]. Recently the Neuropathic Pain Special Interest Group (NeuPSIG) of the International Association for the Study of Pain has updated evidence-based recommendations for pharmacotherapy of neuropathic pain [1] using the Grading of Recommendations Assessment, Development, and Evaluation (GRADE) system. Pregabalin, gabapentin, serotonin and noradrenaline reuptake inhibitor (SNRI) antidepressants particularly duloxetine, and tricyclic antidepressants (TCAs) received strong GRADE recommendations for use and are recommended as first-line for peripheral neuropathic pain, although with caution as regards TCAs. Capsaicin high concentration patches, lidocaine patches and tramadol received weak GRADE recommendation for use and are recommended as generally second line for peripheral neuropathic pain and local pain generator. Strong opioids and botulinum toxin type A (BTX-A received weak GRADE recommendations for use mainly because of efficacy in most trials but safety concerns (opioids) or lower quality of evidence (BTX-A). These drugs are recommended as third line by specialist use as regards BTX-A. There was a weak GRADE recommendation against the use of oromucosal cannabinoids (Sativex) and valproate and strong GRADE recommendations against the use of levetiracetam and mexiletine because of generally negative results and safety concerns (mexiletine). Other drug treatments (e.g. other antiepileptics, antidepressants and topical treatments, tapentadol and NMDA antagonists) or combination therapy received inconclusive GRADE recommendations because of generally discrepant findings, although some of these drugs might be effective in subgroups of patients. These drugs can be recommended in familial amyloid neuropathy, but topical agents have a major advantage in this context because of their low risk of systemic side effects.
